# Assessment of conbercept therapy for high myopia macular neovascularization by optical coherence tomography angiography

**DOI:** 10.1038/s41598-020-74073-1

**Published:** 2020-10-12

**Authors:** Lu-Na Cheng, Yu-Xi Lin, Lei Liu, Xu-He Zhang, Yan-Qi Xue, Sheng-Di Zhou, Zhe-Li Liu, Han Zhang

**Affiliations:** 1 Department of Ophthalmology, The First Hospital of China Medical University, 155 Nanjing North Street, Heping District, Shenyang, 110001 China; 2 Department of Public Service, The First Hospital of China Medical University, Shenyang, 110001 China

**Keywords:** Retinal diseases, Drug therapy, Medical imaging

## Abstract

This study aimed to evaluate the efficacy and safety of the intravitreal injection of conbercept in the treatment of macular neovascularization (MNV) secondary to high myopia and to observe the application of optical coherence tomography angiography (OCTA) in the treatment follow-up. We reviewed the medical records of 20 patients (21 eyes) with MNV secondary to high myopia who were enrolled in the Department of Ophthalmology of the First Hospital of China Medical University between May 2018 and January 2020. Each patient received one or more intravitreal injections of conbercept (0.5 mg/0.05 mL). The treatment was conducted according to a 1 + PRN (*pro re nata*) regimen. The changes of best corrected visual acuity (BCVA), central macular thickness (CMT), and selected MNV and flow areas measured by OCTA were observed over a 6-month follow-up period. The mean logarithm of the minimum angle of resolution (logMAR) BCVA was 1.03 ± 0.61 before treatment and improved to 0.83 ± 0.59 (*P* = 0.007), 0.78 ± 0.62 (*P* = 0.001), 0.81 ± 0.73 (*P* = 0.027), and 0.79 ± 0.72 (*P* = 0.023) at 1 month, 2 months, 3 months, and 6 months after treatment, respectively. The mean CMT was 358.16 ± 206.11 μm before treatment and decreased to 295.38 ± 178.70 μm (*P* = 0.003), 288.34 ± 165.60 μm (*P* = 0.004), 284.36 ± 163.07 μm (*P* = 0.005), and 283.00 ± 160.32 μm (*P* = 0.004) at 1 month, 2 months, 3 months, and 6 months after treatment, respectively. Nineteen eyes (90.5%) had stable or improved vision at 6 months of follow-up. One month after conbercept injection, in OCTA images, the small-diameter blood vessels of the MNV decreased, the intertwined small blood vessels decreased or even disappeared, and the main or larger-diameter blood vessels were still present. The mean selected MNV and blood flow areas were 0.62 ± 0.81 and 0.22 ± 0.27 mm^2^, respectively, before treatment and decreased to 0.23 ± 0.33 and 0.07 ± 0.08 mm^2^ (*P* = 0.04 for both), respectively, 1 month after treatment. No drug-related systemic or ocular adverse effects were observed. Our results suggest that conbercept can effectively and safely improve BCVA and reduce CMT in patients with myopic MVN (mMNV). OCTA can be used to observe MNV area, blood flow area, and MNV morphological changes after treatment with conbercept, thus providing a reference for treatment follow-up.

## Introduction

Myopia is the most common refractive error, and complications of high myopia are among the main causes of irreversible vision loss worldwide. Myopia is especially common in Asian populations, with prevalence rates of 9–21%, compared with 2–4% in Caucasians^1,2^. Related research reveals that the prevalence of myopia and high myopia will continue to increase. It is estimated that by 2050, the prevalence of myopia in the world will be the highest in the high-income region in the Pacific (66.4%) and East Asia (65.3%) and the lowest in East Africa (22.7%) and Oceania (23.8%)^3^. High myopia is often accompanied by various pathological changes in the posterior pole of the eye, including sclera thinning, choroidal atrophy, macular hole, retinal fissure, retinal detachment, Fuchs spots, lacquer cracks, retinal degeneration, macular neovascularization (MNV), and others^4^. Myopic macular neovascularization (mMNV) occurs in 5–10% of high myopic patients and is the leading cause of irreversible vision loss in working people aged less than 50 years. The vision loss is mainly due to the development of chorioretinal atrophy and increased deterioration of the retinal pigment epithelium (RPE) near regressed MNV^5^.

At present, there are many clinical methods for detecting MNV, such as fundus fluorescein angiography (FFA) and indocyanine green angiography (ICGA). However, FFA and ICGA require intravenous injection, which is time-consuming and may cause serious complications, such as allergic reactions. Noninvasive optical coherence tomography (OCT) and optical coherence tomography angiography (OCTA) play unique roles in the diagnosis and follow-up of mMNV^6^.

Vascular endothelial growth factor (VEGF) plays a key role in the development of various types of MNV, including mMNV^7^. Therefore, intravitreal injection of anti-vascular endothelial growth factor (anti-VEGF) drugs has become an effective method for the treatment of mMNV. Intravitreal antiangiogenic therapy has been proven to be effective in treating mMNV, not only by improving the acute symptoms but also by potentially preventing the development of chorioretinal atrophy^8^.

Conbercept (Chengdu Kanghong Biotech Ltd., Sichuan, China) is a soluble recombinant VEGF receptor that can competitively bind all VEGF-A isoforms, VEGF-B, and placental growth factor (PlGF). Conbercept has been proven safe and effective for treating MNV^9^. However, there are few reports on the efficacy of conbercept in treating mMNV. Our study investigated the efficacy and safety of conbercept in the treatment of mMNV and the changes of MNV during follow-up with the use of OCTA.

## Results

Twenty patients (21 eyes) with mMNV were included in the study. The baseline characteristics of study patients are presented in Table 1. There were 18 eyes from 18 women and 3 eyes from 2 men. The mean patient age at the start of the study was 58.95 ± 12.28 years. The mean spherical equivalent refractive error was − 11.53 ± 4.28 D. Before treatment, the mean logarithm of the minimum angle of resolution (logMAR) best corrected visual acuity (BCVA) was 1.03 ± 0.61 (20/214 in Snellen equivalent) and the mean central macular thickness (CMT) was 358.16 ± 206.11 μm.

**Table 1:** Demographic and baseline clinical features of patients with myopic macular neovascularization.

Feature	Value
Age (years), mean ± SD	59.0 ± 12.3
**Sex, n (%)**	
Male	18 (90.0)
Female	2 (10.0)
Refractive error (diopters), mean ± SD	− 11.5 ± 4.3
BCVA (logMAR), mean ± SD	1.03 ± 0.61
CMT (μm), mean ± SD	358.16 ± 206.11
MNV area (mm^2^), mean ± SD	0.62 ± 0.81
Blood flow area (mm^2^), mean ± SD	0.22 ± 0.27

*BCVA* best-corrected visual acuity, *CMT* central macular thickness, *LogMAR* logarithm of minimal angle of resolution, *MNV* macular neovascularization, *SD* standard deviation.

**Figure 1:**
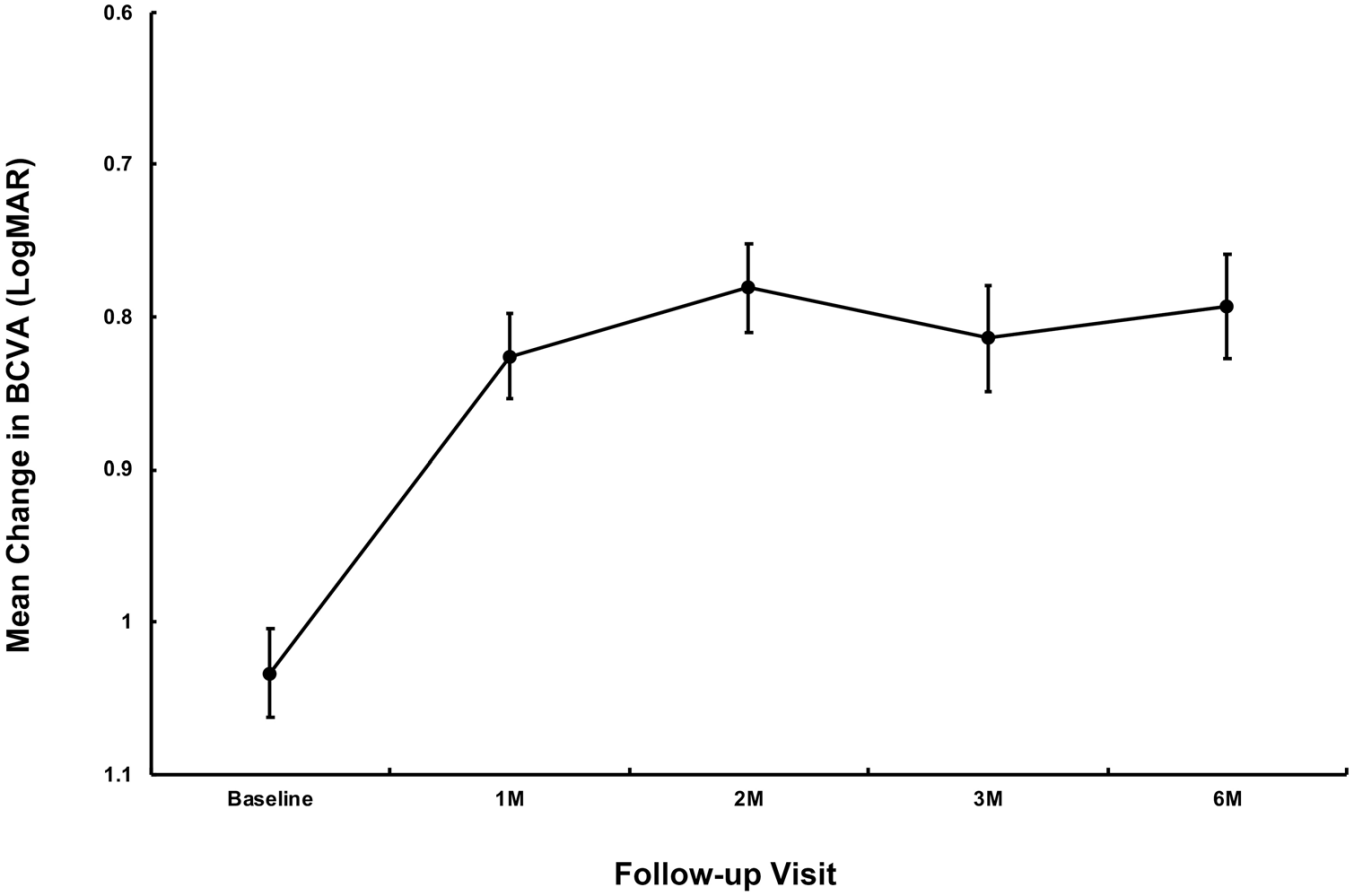
Variations in mean logarithm of minimal angle of resolution (logMAR) best-corrected visual acuity (BCVA) over a period of 6 months after intravitreal conbercept for myopic macular neovascularization (mMNV) treatment. The standard error of the mean is indicated by error bars.

The mean logMAR BCVA at 1 month, 2 months, 3 months, and 6 months after treatment was 0.83 ± 0.59 (20/135 in Snellen equivalent) (*P* = 0.007), 0.78 ± 0.62 (20/121 in Snellen equivalent) (*P* = 0.001), 0.81 ± 0.73 (20/129 in Snellen equivalent) (*P* = 0.027), and 0.79 ± 0.72 (20/123 in Snellen equivalent) (*P* = 0.023), respectively. The changes were statistically significant compared with baseline (Fig. 1). At the final follow-up, 13 of the 21 eyes (61.9%) exhibited an improvement, 6 eyes (28.6%) remained unchanged, and 2 eyes (9.5%) had a deterioration.

**Figure 2:**
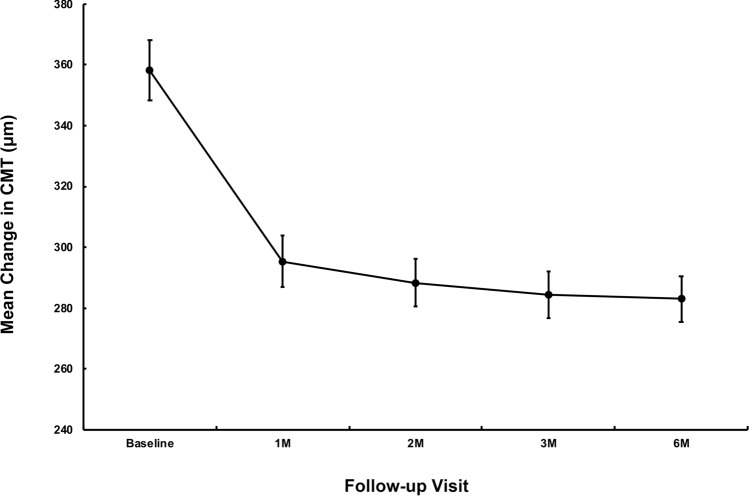
Variations in mean central macular thickness (CMT) over 6 months after intravitreal conbercept for myopic macular neovascularization (mMNV) treatment. The standard error of the mean is indicated by error bars.

The mean CMT at 1 month, 2 months, 3 months, and 6 months after treatment was 295.38 ± 178.70 μm (*P* = 0.003), 288.34 ± 165.60 μm (*P* = 0.004), 284.36 ± 163.07 μm (*P* = 0.005), and 283.00 ± 160.32 μm (*P* = 0.004), respectively. The changes were statistically significant compared with baseline (Fig. 2).

**Figure 3:**
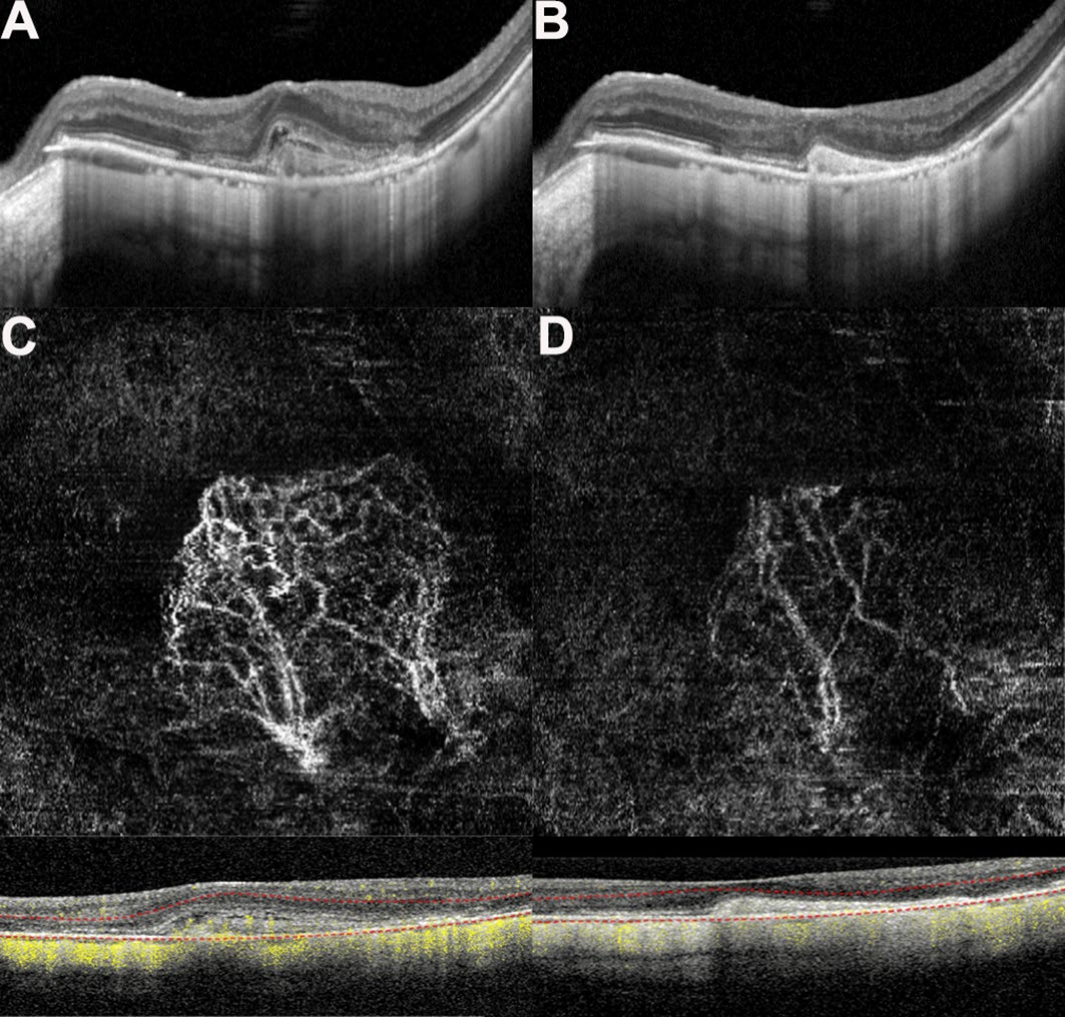
(**A**,**B**) Optical coherence tomography (OCT) images of a patient with myopic macular neovascularization (mMNV) before and after receiving conbercept. Central macular thickness (CMT) decreased from 371.43 to 248.98 μm. The hyperreflective signal lesions in the outer layer of the retina shrank and the outer structure tended to normalize. (**C**,**D**) Optical coherence tomography angiography (OCTA) images of the patient before and after injection. (**C**) Presents a large-area, highly organized, interlaced high-flow MNV vascular network. It contains small capillary branches and nourishing blood vessels and is surrounded by a low-signal halo, showing a leafy tree crown or sea fan shape. One month after conbercept treatment, the MNV of (**D**) shows only nourishing blood vessels with larger diameters, the small blood vessels decreased or even disappeared, the blood vessels intertwined, and the blood flow was significantly reduced. The MNV areas in (**C**) and (**D**) are 2.69 and 1.08 mm^2^, while the blood vessel areas are 0.92 and 0.24 mm^2^, respectively.

**Figure 4:**
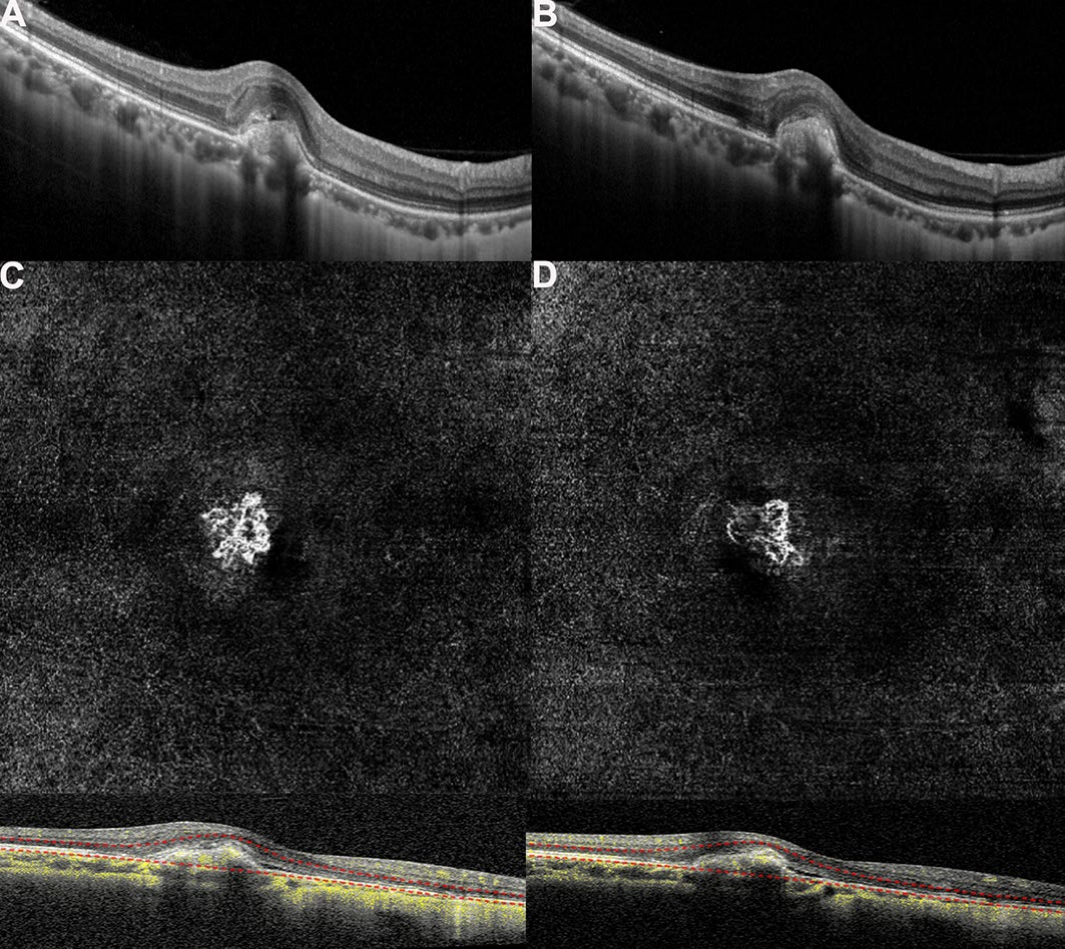
(**A**,**B**) Optical coherence tomography (OCT) images of a patient with myopic macular neovascularization (mMNV) before and after receiving conbercept. Central macular thickness (CMT) decreased from 281.63 to 261.22 μm, and the hyperreflective signal lesions in the outer layer of the retina decreased. (**C**,**D**) Optical coherence tomography angiography (OCTA) images of the patient. MNV in both figures shows a small, vascular chaotic high-flow vascular network with a tree bud shape. The MNV areas in (**C**) and (**D**) are 0.54 and 0.23 mm^2^, while the blood vessel areas are 0.23 and 0.13 mm^2^, respectively.

Before treatment, the morphological shape of the MNV in OCTA images was like a flower, fan, or small tree bud. Two subtypes of myopic MNV were distinguished^10,11^ on the basis of different characteristics in size and morphology: the well-organized larger “interlacing” pattern and the small, disorganized MNV “vascular loop” subtype (Figs. 3, 4). The intraobserver agreement was 95.2%. After conbercept injection, the small-diameter blood vessels of the MNV decreased, the intertwined small blood vessels decreased or even disappeared, and the main or larger-diameter blood vessels were still present, but blood flow was significantly reduced. The OCT and OCTA changes in a patient is shown in Figs. 3 and 4. The mean selected MNV area and blood flow area were 0.62 ± 0.81 and 0.22 ± 0.27 mm^2^, respectively, before treatment, and decresed to 0.23 ± 0.33 and 0.07 ± 0.08 mm^2^ (*P* = 0.04 for both), respectively, 1 month after conbercept treatment (Table 2).

**Table 2:** Comparison of measurements between baseline and 1 month after conbercept treatment in myopic macular neovascularization.

Feature	Baseline	1-month posttreatment	*P* value
BCVA (logMAR), mean ± SD	1.03 ± 0.61	0.83 ± 0.59	0.007
CMT (μm), mean ± SD	358.16 ± 206.11	295.38 ± 178.70	0.003
MNV area (mm^2^), mean ± SD	0.62 ± 0.81	0.23 ± 0.33	0.04
Blood flow area (mm^2^), mean ± SD	0.22 ± 0.27	0.07 ± 0.08	0.04

*BCVA* best-corrected visual acuity, *CMT* central macular thickness, *LogMAR* logarithm of minimal angle of resolution, *MNV* macular neovascularization, *SD* standard deviation.

Among the patients, 49 intravitreal injections were administered in total during the 6-month study period (mean = 2.33 injections per eye). Nineteen of the 21 eyes (90.48%) required reinjections (range 2–4 injections), of which 12, 5, and 2 eyes needed 2, 3, and 4 injections, respectively.

During treatment and follow-up, there were no serious complications or adverse reactions, such as endophthalmitis, retinal detachment, and stroke.

## Discussion

High myopia is the second leading cause of MNV next to neovascular age-related macular degeneration (nAMD) and the main cause of irreversible vision loss in people below 50 years of age. Between 5 and 11% of people with myopia will develop mMNV^12^. Without treatment, the prognosis for mMNV is poor, with over 90% of affected eyes likely to have a progressive and irreversible deterioration of vision leading to blindness within 10 years^12,13^. Reportedly, 35% of patients with preexisting mMNV developed MNV in the fellow eye, and the mean period between the development of MNV in the first eye and that in the second eye was 8 years^14^.

There are many methods for treating mMNV, including thermal laser photocoagulation, photodynamic therapy, transpupillary thermotherapy, and surgical intervention. However, long-term follow-up studies have found that the prognosis is poor, and the curative effects of these treatments are not satisfactory^12^. Recently, anti-VEGF drugs have become the primary treatment for mMNV. At present, anti-VEGF drugs include bevacizumab, ranibizumab, aflibercept, and conbercept. Large, multicenter phase III studies (RADIANCE, BRILIANCE, and MYRROR) have confirmed the effectiveness of ranibizumab^15,16^ and aflibercept^17^ in treating mMNV. Conbercept (Chengdu Kanghong Biotech Ltd., Sichuan, China) is an anti-VEGF fusion protein, binding to all isoforms of VEGF-A and to PlGF and VEGF-B. It contains the second immunoglobulin-like domain of vascular endothelial growth factor receptor 1 (VEGFR-1), the third and fourth Ig-like domains of VEGFR-2, and the Fc fragment of human IgG produced by the Chinese hamster ovary (CHO) cell expression system^18^. Compared with aflibercept, conbercept has a higher affinity for VEGF due to the addition of a fourth Ig-like domain of VEGFR-2 in the Fab region^18^. Additionally, PlGF binds to VEGFR-1 to promote chemoattraction and aggregation of monocytes and macrophages^19^. Simultaneous inhibition of both VEGF and PlGF suppresses the expression of tumor necrosis factor alpha (TNF-α), interleukin 1 (IL-1), and other inflammatory cytokines, which can alleviate MNV to some extent^20^.

There are few studies on the long-term efficacy of conbercept in treating mMNV. Yang M et al. reported a 12-month follow-up of 42 mMNV eyes treated with conbercept^21^. They revealed that BCVA and CMT were effectively improved from baseline, confirming the effectiveness and safety of conbercept. Results of our study showed that intravitreal conbercept treatment in patients with mMNV was associated with a significant improvement in BCVA and with a marked decrease in CMT during 6 months of follow-up. Compared with baseline, the percentage of stable and improved vision at the last follow-up was 90.5%. Therefore, the results of our study proved that intravitreal injection of conbercept is effective for mMNV. During follow-up observation, no serious complications were found, proving the safety of conbercept for the treatment of mMNV.

Recent clinical application of OCTA has made up for the shortcomings of FFA with its advantages, such as noninvasiveness, rapidness, and reproducibility. OCTA is derived from OCT and provides a layered image of choroid and retinal blood vessels to detect in real time the flow of erythrocytes in blood vessels^22^. Simultaneously, the OCT-B scan presents a direct comparison between the structure and angiogram on the same cross section. MNV in the OCTA image is similar to that in the early ICGA image, but the blood vessel characteristics are clearer. OCTA can easily identify the main blood vessels and the attached branch blood vessels with terminal vessel rings. At the same time, it can better identify MNV types and assess morphological changes after treatment because there are no leakage and accumulation of dyes. In a study by Bruyere et al., one case of mMNV revealed no fluorescence leakage in late FFA, and spectral-domain OCT (SD-OCT) revealed slight subretinal hyperreflective exudation, and it appeared as a small high blood flow neovascularization on OCTA^10^. The study indicated that OCTA was capable of detecting small MNVs.

The results of our study revealed that the average baseline vascular area of mMNV measured by OCTA was 0.62 ± 0.81 mm^2^, and the blood flow area was 0.22 ± 0.27 mm^2^. The vascular area of mMNV of our study is close to the finding by Miyata et al. (0.59 ± 0.56 mm^2^)^23^, which is significantly larger than the finding by Bruyere et al. (0.22 ± 0.27 mm^2^)^10^. Furthermore, we have distinguished two subtypes of mMNV similar to those of Bruyere et al., with different characteristics of size and morphology: a large, highly organized, interlacing high-flow network containing small capillary ramifications, bordered by a dark halo, with feeder vessel (Fig. 3) and a small, disorganized MNV “vascular loop” subtype, showing irregular blood flow and a small tree bud-shaped lesion composed of a small number of discontinuous linear branch blood flows(Fig. 4)^10^.

Previous studies reported the vacular remodeling of MNV after anti-VEGF therapy in nAMD on OCTA^24,25^. In nAMD, the pruning of smaller vessels occurred 24 h after anti-VEGF treatment, increasing and reaching the maximum flow regression between 6 and 12 days, followed by reproliferation (reopening or new sprouting of the vessels) after 20–50 days^24^. In another case series, the authors identified two types of MNV morphology progression in after anti-VEGF treatment: constant patterns, conserving their disorganized morphology with tiny capillaries and loops, are suggestive for immature MNV; however, changing patterns are subject to arteriolization with thicker dilated vascular trunks and the absence of tiny ramifications, ultimately suggestive for a mature neovascular lesion^25^. In patients with mMNV, a study analyzed the 1-month follow-up course after anti-VEGF treatment. mMNV were classified as irregular mass, referred to as tree-in-bud or a nearly round-shaped mass with a continuous ring around the lesion. At 1 month, changes were observed with decreased MNV size, narrowed lesion with the pruning of thinner peripheral blood flow, and decreased network density^6^. Similarly, in our study, the mean blood vessel area was 0.23 ± 0.33 mm^2^ and the mean blood flow area was 0.07 ± 0.08 mm^2^; both values significantly reduced compared with those of baseline (P = 0.04 for both) 1 month after intravitreal conbercept injection. OCTA images revealed that the capillaries and small-caliber feeder vessels were attenuated significantly after 1 month of conbercept treatment, which may have decreased the flow signals or caused these vessels to regress. However, the main central trunk vessel and large-caliber feeder vessels remained unchanged. These findings are similar to those of the study by Huang et al.^26^. Further studies are required to investigate the morphological changes in mMNV on OCTA, before and after treatment with anti-VEGF agents and the correlations between these changes and signs of neovascular activity.

This study had certain limitations. Segmentation errors are not rare in OCTA examinations of eyes with high myopia because a thinner retina causes enhanced visualization of choroidal vessels that are occasionally difficult to differentiate from MNV. In our study, cross-sectional OCTA used in conjunction with en face OCTA and careful manual segmentation were performed to confirm and evaluate the presence of MNV. However, segmentation errors and enhanced visualization of choroidal vessels can also affect the detection and evaluation of MNV. Other limitations include that the sample size was small, observation period was short, and there was no control group. The conclusions need to be confirmed by expanding the sample size, extending the follow-up time, and setting up a control group.

According to the results of our study, patients with mMNV treated with conbercept had improved BCVA, reduced CMT, reduced MNV area, and reduced blood flow area and did not have serious adverse reactions. Therefore, conbercept is effective and safe for the treatment of mMNV. During the follow-up, OCTA provided sensitive and intuitive images and quantitative analysis to monitor and evaluate the therapeutic effect of intravitreal anti-VEGF for mMNV.

## Methods

The study was a retrospective analysis of a case series. We reviewed the medical records of 20 patients (21 eyes) with MNV secondary to high myopia who were enrolled in the Department of Ophthalmology of the First Hospital of China Medical University between May 2018 and January 2020. The ethics committee and institutional review board of China Medical University First Hospital approved the study protocol, which was conducted in agreement with the Declaration of Helsinki. Written informed consents were obtained from the patients before the treatment.

The inclusion criteria were age > 18 years; diopters >  − 6. 00 D; fundus examination showing high myopia changes, such as lacquer crack and chorioretinal atrophy; OCT examination showing active MNV in the macula (hyper-reflective MNV lesion with fuzzy borders above RPE with or without intraretinal or subretinal fluid); receiving intravitreal conbercept treatment and undergoing 6 months of follow-up. The exclusion criteria were severe cataract or vitreous hemorrhage; age-related macular degeneration, diabetes, or any cause of secondary MNV other than myopia; recent use of anticoagulants; and previously having undergone anti-VEGF therapy, retinal thermal laser photocoagulation, and photodynamic therapy.

All eyes underwent complete ophthalmologic evaluation at baseline, including BCVA testing using a standard Snellen chart, slit-lamp biomicroscopy, IOP measurement, indirect ophthalmoscopy, SD-OCT, and OCTA (SPECTRALIS, Heidelberg Engineering, Heidelberg, Germany).

Three days before treatment, the patients were instructed to use 0.5% levofloxacin drops four to six times a day. At baseline, the patients were given intravitreal injections of 0.5-mg conbercept (0.05 mL). All injections were administered under sterile conditions in the operating room. The eyelids were cleaned using povidone-iodine solution, and a lid speculum was inserted. Subsequently, topical anesthesia was applied, and the conjunctiva was soaked with 5% povidone-iodine. The pars plana was injected with a 30-guage needle and 0.05 mL of conbercept was administered into the vitreous cavity. The next day after treatment, the patients were instructed to use 0.5% levofloxacin drops four to six times a day for 1 week. Follow-up examinations at 1 day, 1 week, and 1 month after the injection were performed; thereafter, they were conducted at monthly intervals over 6 months. At each visit, BCVA testing, slit-lamp examination, IOP measurement, indirect ophthalmoscopy, OCT, and OCTA were performed. Because the avascular complex layer seems to be typical and shows prominent MNV, our OCTA images were collected at the level of the avascular complex layer (between the outer plexiform layer and Bruch’s membrane). The automatic segmentation provided by the OCTA software was manually adjusted by modulating the segmentation lines for correct visualization of the MNV. Manual measurement of the MNV and blood flow areas was performed using ImageJ software (public domain software; National Institutes of Health, Bethesda, Maryland, USA). Briefly, the MNV area was manually outlined using the free-hand selection tool, and its dimension was expressed in squared millimetres. To calculate the blood flow area, each image was converted from 8-bit into red–green–blue (RGB) color type; the image was split into the three channels (red, green, and blue) and the red channel was chosen as the reference. The threshold tool set was adjusted to default and the dark-background option was selected. This tool automatically the set lower and upper threshold values (100–255 in our case, respectively), and segmented greyscale images into features of interest and background. The total vessel area in the MNV lesion was considered as blood flow area.

The treatment was conducted according to a 1 + PRN (*pro re nata*) regimen. Additional reinjections were given at least 4 weeks after the previous injection according to the following criteria: self-reported significant central visual acuity loss and/or worsening symptoms; fundus examination revealing new bleeding; and OCT exudative signs, such as intraretinal and/or subretinal fluid.

The proportion of eyes that had improved (≥ 1 line), stable (within 1 line), or deteriorated (≥ 1 line) vision at the 6-month follow-up was the primary outcome. Changes in mean BCVA and CMT from baseline to 6 months, variations of the selected MNV area and MNV blood flow area by OCTA 1 month after injection, MNV morphological changes, treatment number, and ocular and systemic safety were included in other outcome measures. Two independent investigators (LN.C. and YX.L.) evaluated the morphological characteristics of MNV on OCTA, including MNV shape, MNV core, MNV margin and overall appearance. On the basis of the overall appearance, MNV was classified as mainly ‘interlacing’ or mainly “vascular loop”, as described previously^10,11^. Any discrepancies in the data were resolved through reassessment and discussion with a senior researcher (H.Z.).

The SPSS version 20.0 (SPSS, Inc., Chicago, IL) was used to perform statistical analysis. Snellen visual acuity was converted to logMAR equivalent for statistical analysis. Data were expressed as mean ± standard deviation. All data were tested for normality using histogram graphical analysis and Kolmogorov–Smirnov numerical analysis. The paired t test was used to compare normally distributed continuous variables. For the comparison of data that were not normally distributed, the Wilcoxon signed rank test was used. P-values < 0.05 were considered statistically significant.
